# Micro Magnetic Field Produced by Fe_3_O_4_ Nanoparticles in Bone Scaffold for Enhancing Cellular Activity

**DOI:** 10.3390/polym12092045

**Published:** 2020-09-08

**Authors:** Shizhen Bin, Ailun Wang, Wang Guo, Li Yu, Pei Feng

**Affiliations:** 1 Research Institute of Light Alloys, Central South University, Changsha 410083, China; shizhenbin@csu.edu.cn (S.B.); walwlz@csu.edu.cn (A.W.); 2 State Key Laboratory of High Performance Complex Manufacturing, College of Mechanical and Electrical Engineering, Central South University, Changsha 410083, China; guowang@csu.edu.cn (W.G.); yujiali@csu.edu.cn (L.Y.)

**Keywords:** magnetic stimulation, Fe_3_O_4_ nanoparticles, cellular activity, selective laser sintering, scaffold

## Abstract

The low cellular activity of poly-l-lactic acid (PLLA) limits its application in bone scaffold, although PLLA has advantages in terms of good biocompatibility and easy processing. In this study, superparamagnetic Fe_3_O_4_ nanoparticles were incorporated into the PLLA bone scaffold prepared by selective laser sintering (SLS) for continuously and steadily enhancing cellular activity. In the scaffold, each Fe_3_O_4_ nanoparticle was a single magnetic domain without a domain wall, providing a micro-magnetic source to generate a tiny magnetic field, thereby continuously and steadily generating magnetic stimulation to cells. The results showed that the magnetic scaffold exhibited superparamagnetism and its saturation magnetization reached a maximum value of 6.1 emu/g. It promoted the attachment, diffusion, and interaction of MG63 cells, and increased the activity of alkaline phosphatase, thus promoting the cell proliferation and differentiation. Meanwhile, the scaffold with 7% Fe_3_O_4_ presented increased compressive strength, modulus, and Vickers hardness by 63.4%, 78.9%, and 19.1% compared with the PLLA scaffold, respectively, due to the addition of Fe_3_O_4_ nanoparticles, which act as a nanoscale reinforcement in the polymer matrix. All these positive results suggested that the PLLA/Fe_3_O_4_ scaffold with good magnetic properties is of great potential for bone tissue engineering applications.

## 1. Introduction

Poly-l-lactic acid (PLLA) has become one of the main bone scaffold materials due to its advantages of good biocompatibility and easy processing [1,2,3]. Nevertheless, the low cellular activity limits its application in bone tissue engineering due to the lacking of active functional groups and weak cell affinity [4,5,6,7]. For enhancing cellular activity, researchers have added various cell growth factors, such as bone morphogenetic protein (BMP), transforming growth factor-beta (TGF-β), fibroblast growth factor, and so on [8,9,10,11]. Schofer et al. [8] incorporated BMP-2 into PLLA nanofibers and found that the BMP-2 improved the scaffold’s cellular activity by increasing the expression of osteogenic marker proteins and osteogenesis. Zhu et al. [9] added TGF-β1 to nano-HA/PLLA composite scaffold and found that TGF-β1 released and promoted the adhesion, spreading, proliferation of mesenchymal stem cells (MSCs). Although the growth factors can improve cellular activity, they are very expensive and have a decay half-life [12,13,14]. The fast decay rate makes its biological activity decrease quickly and it cannot be continuously and steadily enhanced, which has greatly limited their wide range of clinical applications.

In recent years, researches have shown that physical stimulation, especially magnetic stimulation, can continuously stimulate cell growth and proliferation, and thus it can be used as an alternative method to increase the cellular activity of bone scaffold [15,16,17]. Due to its unique magnetic properties, Fe_3_O_4_ nanoparticles have been receiving considerable interest in biomedical applications [18,19,20]. When the particle size is less than 30 nm in diameter, the thermal fluctuation energy is equivalent to the magnetocrystalline anisotropy energy, which is enough to cause the whole crystallite to rotate freely, thereby exhibiting superparamagnetism [21,22]. Even if no external magnetic field is applied, the superparamagnetic Fe_3_O_4_ nanoparticle can be regarded as a single magnetic domain without a domain wall, providing a micro-magnetic source for the nano-scale magnetic field [23,24]. In addition, Fe_3_O_4_ nanoparticles approved by the Food and Drug Administration (FDA) of United States of America for clinical use, have excellent biocompatibility and safety, and are widely used in bone tissue engineering [25,26,27]. Taking into account the above characteristics, we suppose that the incorporation of superparamagnetic Fe_3_O_4_ nanoparticles into the bone scaffold makes it possible for them to generate a large number of tiny magnetic fields in the scaffold, which can activate and enhance cell activity continuously and steadily through magnetic stimulation.

At present, there have been some reports on the research of Fe_3_O_4_ nanoparticles to enhance cell activity [28,29,30,31]. For example, Shan et al. [28] reported that the incorporation of Fe_3_O_4_ nanoparticles into PLLA fibers enhanced cell adhesion and proliferation without significant cytotoxicity. Wu et al. [29] integrated Fe_3_O_4_ nanoparticles into CaP bioceramics and found that magnetic fields generated by Fe_3_O_4_ nanoparticles enhanced the activity of ALP and promoted the differentiation and proliferation of osteoblasts. Wei et al. [30] also indicated that the introduction of magnetic Fe_3_O_4_ nanoparticles into the CS/PVA fiber membrane could promote cell proliferation and accelerate the bone-like tissue formation. However, all the above studies were basically mainly concentrated on composite materials or fibers, and there were few studies on incorporating Fe_3_O_4_ nanoparticles into scaffolds for bone regeneration.

In this study, Fe_3_O_4_ nanoparticles were incorporated into PLLA scaffold via selective laser sintering (SLS) to continuously and stably enhance the cellular activity. The phase composition, thermal properties, magnetic properties, and mechanical properties of the PLLA/Fe_3_O_4_ scaffolds were comprehensively studied. The effects of the magnetic composite scaffolds on the adhesion, proliferation, and differentiation of MG63 cells are studied, discussed, and explained.

## 2. Materials and Methods

### 2.1. Materials

PLLA powders (average particle size: 0.2–5 µm) were purchased from Jinan Daigang Biomaterial Co., Ltd. (Jinan, China). Fe_3_O_4_ nanoparticles (average particle size: 20 nm) were kindly provided by Shanghai Jichun Industrial Co., Ltd. (Shanghai, China). Dulbecco’s Modified Eagle Medium (DMEM), phosphate buffer solution (PBS) and fetus bovine serum (FBS) were purchased from Biological Industries (Beit HaEmek, Israel). MG63 cells were provided by American Type Culture Collection (Manassas, VA, USA). CCK-8 solution was bought from Selleck Chemicals (Houston, TX, USA). All other reagents were purchased from Shanghai Macklin Biochemical Technology Co., Ltd. (Shanghai, China).

### 2.2. Scaffold Preparation

**Figure 1:**
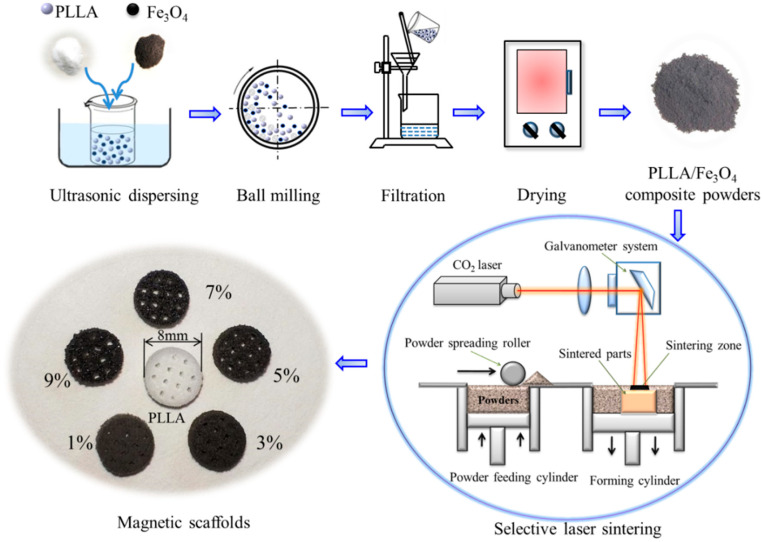
A schematic of the preparation of PLLA/Fe_3_O_4_ magnetic composite scaffolds.

The preparation process of PLLA/Fe_3_O_4_ magnetic composite scaffolds included the preparation of composite powders and scaffolds, as schematically depicted in Figure 1. First, the PLLA and Fe_3_O_4_ powders were weighed in a certain weight ratio (Fe_3_O_4_ content in the composite: 0, 1, 3, 5, 7, and 9 wt %). Then, the two powders were added into a beaker containing 50 mL of absolute ethanol, and then the mixed solution was ultrasonically dispersed for 30 min. After that, the mixed solution was poured into a ball mill for 1 h for further dispersing. Finally, PLLA/Fe_3_O_4_ composite powders were obtained by filtering the mixed solution slowly using a filter paper with pore size of 0.45 µm (Millipore, HAWP01300) and drying in a drying box for 24 h at 50 °C.

The magnetic composite scaffold was prepared by a SLS system with a 100 W CO_2_ laser (λ = 10.6 μm) and a galvanometric scanning system. In detail, the powder feeding cylinder piston rises, and then the powder spreading roller evenly lays a layer of powder on the sintering platform. Then under the control of the galvanometric scanning system, the powder layer was scanned and sintered by a laser beam followed the cross-sectional profiles of the model [32]. After sintering a layer, the piston of the forming cylinder was lowered by one layer thickness. Then, the powder spreading roller was controlled to lay a new layer of powder above the previously sintered layer, followed by the next sintering of the powder. The above operation was repeated in this way, and the sintered layers were stacked layer by layer until the whole scaffold was formed. The main process parameters were optimized as follows: scanning speed of 180 mm s^−1^, scanning interval of 0.15 mm, and layer thickness of 0.1 mm. Six kinds of PLLA/Fe_3_O_4_ scaffolds with different contents of Fe_3_O_4_ (0, 1, 3, 5, 7, and 9 wt %) were fabricated by SLS, as shown in Figure 1. The optical color of the scaffolds gradually deepens with the increase of Fe_3_O_4_ content.

### 2.3. Characterization

The phase constituent of Fe_3_O_4_ nanoparticles and magnetic scaffolds was investigated via XRD (DMAX 2500, Japan Science Co., Tokyo, Japan) at a scan rate of 8°/min in the range of diffraction angle 2θ = 10°~80°. The chemical group analysis was performed by FTIR (Nicolet 6700, Thermo Electron Scientific Instruments Co., Madison, WI, USA) with a test wavelength of 500 to 4000 cm^−1^ and a number of scans of 16 times. The TGA and DSC curves of the magnetic scaffolds at 30 to 600 °C were measured to evaluate the thermal stability, using a thermo gravimetric analyzer (TGA-105, Nanjing Dazhan Electromechanical Technology Research Institute, Nanjing, China) under nitrogen at a temperature rise rate of 20 °C/min. Magnetic properties of the magnetic composite scaffolds were detected by a vibrating sample magnetometer (VSM7407, Lake Shore Cryotronics Inc., Westerville, OH, USA) and a permanent magnet. The hysteresis loop was measured in an applied magnetic field of ±20 kOe and the saturation magnetization was evaluated.

The compressive strength and modulus were evaluated using a universal testing machine (WD-D1, Shanghai Zhuoji Instrument Co., Ltd., Shanghai, China). The force-displacement curve was recorded automatically by a flat indenter with a slow loading speed of 0.5 mm/min. The compression strength and elastic modulus of the sample was calculated from the compression stress-strain curve. The hardness of composite scaffolds was assessed by a digital micro Vickers hardness tester (Micro Vickers Hardness Tester, HVS-1000C Shenzhen Shunhua Instrument Equipment Co., Ltd., Shenzhen, China) using an indentation test after polished. The Vickers hardness was calculated by the equation [33]: *HV* = 0.1891*F*/*d*^2^, where *F* is the test force (N) and *d* is the diagonal length (mm). Each set of data was averaged and standard deviation from five replicate samples. The microscopic morphology of the surface pores and sections of the scaffold were characterized by scanning electron microscopy (SEM, Phenom ProX, Phenom-World BV, Eindhoven, Netherlands).

### 2.4. Cellular Compatibility

MG63 cells were cultured to evaluate the cell compatibility of scaffolds owning to their similar matrix synthesis and mineralization capabilities to osteoblasts. The cells were cultured in DMEM supplemented with sodium pyruvate, 10% FBS, 100 U/mL penicillin and 100 μg/mL streptomycin at 37 °C in a humidified 5% CO_2_ atmosphere. The magnetic scaffold was sterilized with an ultraviolet lamp for 2 h and then placed in a 24 well culture plate for evaluation of cell adhesion and proliferation. MG63 cells were seeded at a density of 4 × 10^5^ cells per well and the cultured medium was changed daily. After 1, 3, and 7 days of culture, the cell-scaffold samples were taken out, rinsed with PBS, immobilized using 4% glutaraldehyde for 30 min, dehydrated with ethanol for 24 h, and completely dried. After being sputtered with gold, the morphology of the cells on the scaffolds was observed by SEM. At each evaluation period, after the cells were stained with 2 μM calcein acetoxymethyl ester for 30 min, the fluorescence microscope equipped with a digital camera was used for observation and analysis.

The CCK-8 method was used to evaluate the proliferation of cells planted on the scaffolds. After 1.0 × 10^4^ MG63 cells were planted on the scaffold and cultured for different days, 40 μl of CCK-8 solution was added to each well and incubated for 4 h, and the absorbance at 450 nm was measured by a microplate reader. The biological activity of the magnetic scaffolds was evaluated by evaluating the degree of differentiation of the cells by detecting the activity of alkaline phosphatase in the medium solution of the scaffold and osteoblasts. After the induction of MG63 cells for 3, 5, and 7 days, the scaffolds were taken out, washed with PBS. The cells were separated by 0.25% trypsin, transferred to a new 24 well plate medium, and washed three times with PBS. After fixing with formalin for 30 s and washing twice with deionized water, they were stained with ALP reagent for 1 h, and finally photographed by a microscope (TE2000U, Nikon Co., Tokyo, Japan).

### 2.5. Statistical Analysis

The quantitative data were expressed as mean ± standard error. The statistical difference was analyzed using student’s t-test and *p* < 0.05 was considered as the level of significance, which is expressed as *.

## 3. Results and Discussion

### 3.1. Physicochemical Properties and Thermal Properties

**Figure 2:**
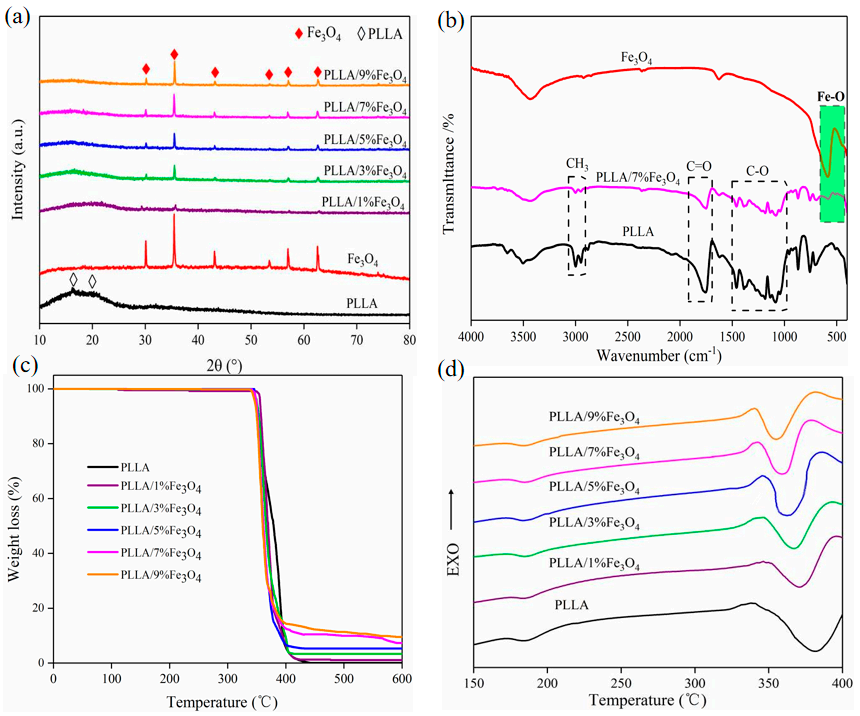
Physicochemical properties and thermal properties of the magnetic scaffolds with 0–9 wt % of Fe_3_O_4_ content. (**a**) XRD patterns. (**b**) FTIR patterns. (**c**) TGA profiles. (**d**) DSC profiles.

The phase composition of the scaffold was analyzed using XRD (Figure 2a). PLLA shows two broad diffraction peaks at 16.5° and 19.9°, indicating that it was a semi-crystalline structure. Fe_3_O_4_ shows diffraction peaks at 30.1°, 35.4°, 43.1°, 53.5°, 57.0°, and 62.6°, which correspond to the crystal planes (220), (311), (400), (422), (511), and (440) [34]. These characteristic peaks of Fe_3_O_4_ were detected in the PLLA/Fe_3_O_4_ composite scaffold, and their intensity increased with the increase of its content, which confirmed that Fe_3_O_4_ was successfully introduced into the scaffold. Compared with the pure PLLA scaffold, the peak of PLLA in the composite scaffold was significantly weakened or even disappeared. This may be because the diffraction peak of Fe_3_O_4_ was too strong and the relative peak intensity of PLLA was weakened. In addition, the positions of the diffraction peaks of PLLA and Fe_3_O_4_ in the composite scaffold did not change, and no other peaks were observed, indicating that SLS preparation did not cause the formation of new phases or phase transformations.

The chemical functional groups of the scaffold were analyzed using FTIR (Figure 2b). PLLA has characteristic absorption peaks at 3000, 1758, and 1500–1000 cm^−1^, which correspond to the stretching vibration peaks of alkyl, carbonyl, and ether groups, respectively [35]. Fe_3_O_4_ has a characteristic absorption peak at 585 cm^−1^, which corresponds to the stretching vibration peak of Fe-O [36]. This characteristic peak was also detected in the PLLA/7%Fe_3_O_4_ magnetic stent, which confirmed the successful introduction of Fe_3_O_4_ again. At the same time, several characteristic peaks of PLLA were clearly detected in the composite scaffold, which confirmed the existence of PLLA and made up for the results of XRD. The thermal stability of the composite scaffold was analyzed using DSC-TGA (Figure 2c,d). The magnetic scaffold exhibits significant thermal weight loss at 335~425 °C (Figure 2c), which was due to the thermal decomposition of PLLA [37]. After 425 °C, the residual weight of the scaffold hardly changed, which was due to the residual Fe_3_O_4_ with high thermal stability (melting point 1594.5 °C). The residual weight was about 0%, 1.2%, 3.4%, 5.2%, 7.6%, and 9.5%, respectively for PLLA/Fe_3_O_4_ scaffold with 0%, 1%, 3%, 5%, 7%, and 9% content, which was closed to the initial amount of Fe_3_O_4_ added. In the DSC curve, PLLA showed two endothermic peaks at 185.1 and 381.5 °C, which correspond to its melting temperature and decomposition temperature, respectively [30,38]. After Fe_3_O_4_ was added, the peak position at 185.1 °C did not change, indicating that the melting point of the scaffold did not change, but the position of the peak at 381.5 °C shifted slightly to the left, which means the thermal decomposition point decreased slightly. This may be due to the addition of Fe_3_O_4_ nanoparticles acting as a catalyst to accelerate the thermal decomposition of PLLA [39].

### 3.2. Magnetic Properties

**Figure 3:**
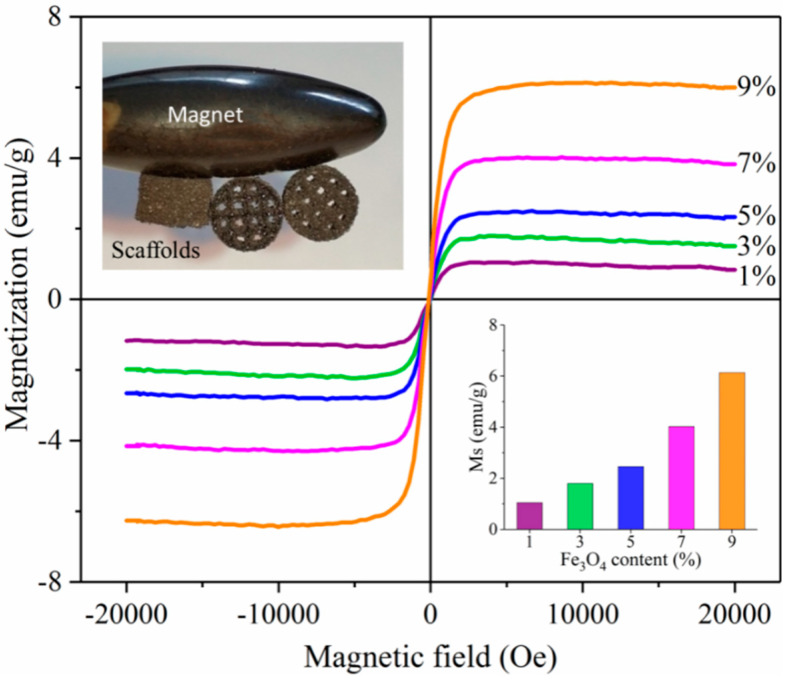
Magnetic properties of composite scaffolds. The main picture was the magnetization curves of the composite scaffolds in the magnetic field at room temperature. The illustration in the upper left corner was an optical view of the PLLA/7%Fe_3_O_4_ composite scaffold attracted by the permanent magnet from different sides. The illustration in the lower right corner was the saturation magnetization (Ms).

The magnetic properties of the composite scaffolds at room temperature were qualitatively and quantitatively evaluated using permanent magnets and Vibrating Sample Magnetometer (Figure 3). As can be seen from the illustration in the upper left corner, the PLLA/7%Fe_3_O_4_ composite scaffold was firmly attracted by the permanent magnet from different sides, showing good magnetic properties. In the applied positive and negative magnetic fields (−20 kOe to +20 kOe), the magnetization curves of the scaffolds passed through the origin and were symmetrical at the origin without magnetization hysteresis, indicating that the scaffolds had good superparamagnetism [28]. The Ms is an extremely important parameter for magnetic performance evaluation, which refers to the maximum magnetization that can be achieved in a magnetic field [22,40]. The Ms of the composite scaffolds calculated from the magnetization curves are shown in the lower right corner of Figure 3. The value of Ms was positively related to the content of Fe_3_O_4_. In detail, the Ms of the 1%, 3%, 5%, 7%, and 9% Fe_3_O_4_ composite scaffolds were 1.1, 1.8, 2.5, 4.0, and 6.1 emu/g. These showed that the composite scaffold had strong magnetism.

### 3.3. Mechanical Properties

**Figure 4:**
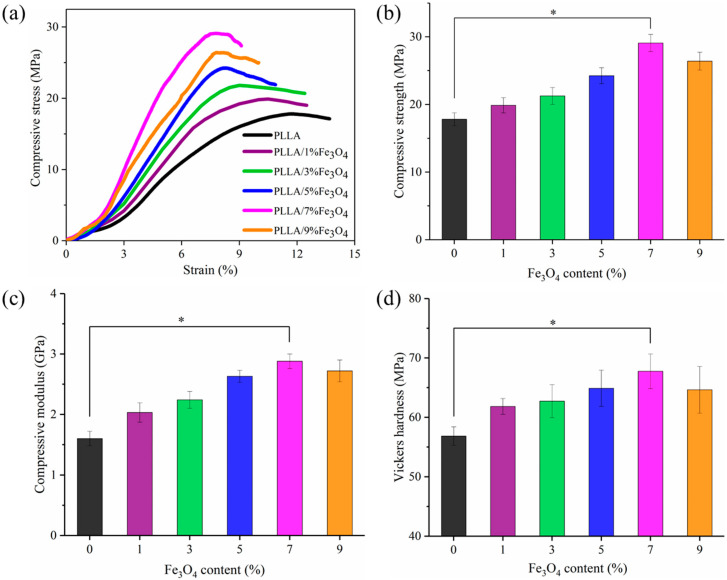
Mechanical properties of the scaffolds. (**a**) Stress-strain curve (**b**) Compressive strength (**c**) Compression modulus (**d**) Vickers hardness. * Represented significant difference (*p* < 0.05) when the PLLA/7%Fe_3_O_4_ scaffold compared with the pure PLLA scaffold.

Mechanical properties were of great importance for use as a bone scaffold because they provided mechanical support in bone repair. The stress-strain curves after the compression test of the scaffolds are shown in Figure 4a. The stress of all the scaffolds increases almost linearly with the strain at the initial stage, and then continues to increase to the maximum peak and then appears an inflection point. The peak was defined as the intensity, and the slope of the initial linear phase was defined as the strength. Then the compressive strength (Figure 4b) and compressive modulus (Figure 4c) were calculated. The compressive strength and modulus in pure PLLA were 17.8 MPa and 1.6 GPa, respectively. After Fe_3_O_4_ was added, the compressive strength of the scaffold was improved. When the content of Fe_3_O_4_ was not more than 7%, the compressive strength increased with the content, and reached the maximum at 7%, which were 29.1 MPa and 2.9 GPa, increased by 63.4% and 78.9%, respectively. Then, when the Fe_3_O_4_ content was further increased to 9%, the compressive strength and modulus decreased compared to 7%, to 26.4 MPa and 2.7 GPa, but were still higher than pure PLLA. The change trend of the Vickers hardness of the scaffolds as a function of with the content of Fe_3_O_4_ was similar to the compression properties, and it reached the optimal value at 7%, which was 67.7 HV (Figure 4d).

**Figure 5:**
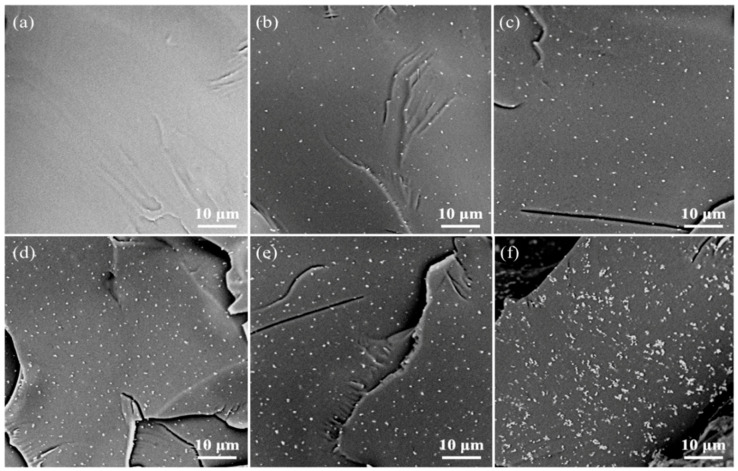
SEM images of fracture surfaces of scaffolds with different proportions. (**a**) PLLA scaffold, (**b**) PLLA/1%Fe_3_O_4_ scaffold, (**c**) PLLA/3%Fe_3_O_4_ scaffold, (**d**) PLLA/5%Fe_3_O_4_ scaffold, (**e**) PLLA/7%Fe_3_O_4_ scaffold, (**f**) PLLA/9%Fe_3_O_4_ scaffold.

The distribution of nano reinforcing phases was closely related to the mechanical properties of polymer nanocomposites. Therefore, the dispersion of Fe_3_O_4_ in the PLLA matrix was analyzed using SEM (Figure 5). The fracture surface of pure PLLA (Figure 5a) was relatively clean and smooth. After adding Fe_3_O_4_, the fracture surface became rough. Fe_3_O_4_ particles were randomly dispersed in the PLLA matrix. When the amount of Fe_3_O_4_ added was no more than 7% (Figure 5e), the number of Fe_3_O_4_ particles on the cross-section increased with the increase of the content of Fe_3_O_4_ added, and a good dispersion was maintained. However, when the Fe_3_O_4_ content was further increased to 9% (Figure 5f), obvious agglomeration began to appear in the scaffold.

Usually, on the premise of uniform dispersion, the more the amount of nanoparticles added, the better the enhancing effect [41]. When the Fe_3_O_4_ content was less than or equal to 7%, the uniformly dispersed Fe_3_O_4_ nanoparticles acted as a nanoscale reinforcement in the polymer matrix and reached a peak at 7%. However, when the content of Fe_3_O_4_ was continuously added to 9%, the excess Fe_3_O_4_ was difficult to uniformly disperse in the matrix, forming more agglomerates, which reduced the enhancement efficiency [42]. Therefore, the mechanical properties of the PLLA/9%Fe_3_O_4_ scaffold no longer continue to increase compared with 7% Fe_3_O_4_.

### 3.4. Cell Responses

**Figure 6:**
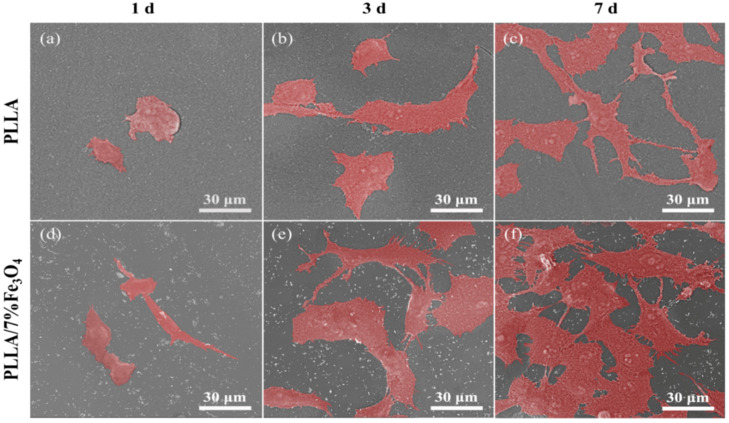
SEM pseudocolor image of MG63 cell adhesion on the scaffolds. (**a**) 1 day on the PLLA scaffold, (**b**) 3 days on the PLLA scaffold, (**c**) 7 days on the PLLA scaffold, (**d**) 1 day on the PLLA/7%Fe_3_O_4_ scaffold, (**e**) 3 days on the PLLA/7%Fe_3_O_4_ scaffold, (**f**) 7 days on the PLLA/7%Fe_3_O_4_ scaffold.

Biocompatibility is very critical for the application of bone scaffolds [43,44]. Based on the previous experimental results, the PLLA/7%Fe_3_O_4_ scaffold with the best comprehensive performance was selected for further culture experiments. The adhesion and morphology of MG63 cells on the scaffolds were characterized by SEM observation (Figure 6). After MG63 cells were cultured on the scaffolds for 1 day, they were spindle-shaped or ellipsoidal. After three days of incubation, the cells expanded on the scaffolds, and obvious filamentous pseudopodia appeared, which helped the cells to adhere tightly to the scaffold and continue to grow. After seven days, the number of cells increased. The cells completely spread out on the surface of the scaffold, and there was a fusion between the cells. On the PLLA/7%Fe_3_O_4_ scaffold, it can be seen that they have been connected to form a fusion layer (Figure 6f). More importantly, MG63 cells exhibited better adhesion morphology and proliferation levels on PLLA/7%Fe_3_O_4_ scaffolds than pure PLLA scaffolds at the same culture time. These showed that the magnetic scaffold had good cell compatibility.

**Figure 7:**
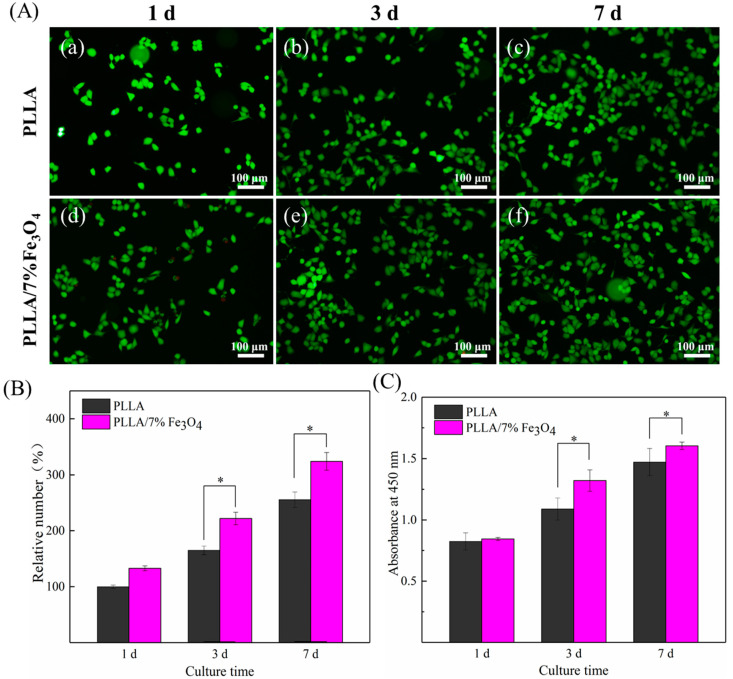
(**A**) Fluorescence image of MG63 cell culture on the scaffolds for 1, 3 and 7 days. (**a**) 1 day on the PLLA scaffold, (**b**) 3 days on the PLLA scaffold, (**c**) 7 days on the PLLA scaffold, (**d**) 1 day on the PLLA/7%Fe_3_O_4_ scaffold, (**e**) 3 days on the PLLA/7%Fe_3_O_4_ scaffold, (**f**) 7 days on the PLLA/7%Fe_3_O_4_ scaffold. (**B**) The relative number of living cells in the fluorescence graph. (**C**) CCK-8 graph.

The behavior of MG63 cells on magnetic bone scaffolds was further studied using fluorescent staining. The results were shown in Figure 7. Obviously, compared with pure PLLA scaffold, there were more green fluorescent cells on magnetic bone scaffolds, and it was positively correlated with the culture time. Taking the number of cells on pure PLLA scaffolds after one day of culture as a 100% comparison, the statistical results are shown in Figure 7B. In order to further study the effect of magnetic scaffolds on the proliferation of MG63 cells, the CCK-8 test was used to evaluate the proliferation capacity of the scaffolds, and the results are shown in Figure 7C. After cell culture for three and seven days, the absorbance value (representing more living cells) on the PLLA/7%Fe_3_O_4_ scaffold was significantly higher than that of the pure PLLA scaffold (*p* < 0.05) indicating that the cell proliferation was enhanced [31]. It was shown that the Fe_3_O_4_ nanoparticles in the scaffolds could obviously promote the proliferation of MG63 cells.

**Figure 8:**
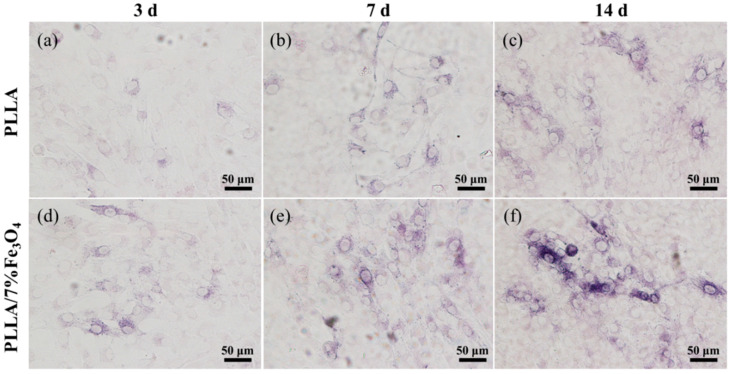
ALP activity staining diagram of MG63 cells on the scaffolds. (**a**) 3 days on the PLLA scaffold, (**b**) 7 days on the PLLA scaffold, (**c**) 14 days on the PLLA scaffold, (**d**) 3 days on the PLLA/7% Fe_3_O_4_ scaffold, (**e**) 7 days on the PLLA/7%Fe_3_O_4_ scaffold, (**f**) 14 days on the PLLA/7% Fe_3_O_4_ scaffold.

ALP is a critical marker for the early differentiation of osteoblasts. Its activity was used to assess the level of differentiation of MG63 cells cultured on magnetic scaffolds (Figure 8). It can be seen from the stained image that the ALP activity increased with increasing culture time. The ALP activity on the magnetic scaffold was higher than the ALP activity on the pure PLLA scaffold, indicating that osteogenic differentiation of MG63 cells was significantly up-regulated, which indicated that the magnetic scaffold had the ability to stimulate MG63 cell differentiation ability.

It is well known that the components contained in the scaffold have a significant effect on the cellular response. Among them, Fe_3_O_4_ nanoparticles have strong magnetic features and unique superparamagnetic properties in nanometric dimensions. Meanwhile, the structure of cell membrane is complex. It not only contains charged lipid molecules, water, and protein, but also contains many ion channels such as K^+^, Na^+^, Ca^2+^ and so on. There is also a large amount of Cl^−^, K^+^, Na^+^, and other anions and cations on the inner and outer surfaces of the membrane [45,46,47]. Therefore, Fe_3_O_4_ nanoparticles can serve as a magnetic source for a single magnetic nanofield in a weak electromagnetic field formed by the cell due to the difference between internal and external ions and charges, thereby generating a biological effect of magnetic field on cells [48,49,50]. Maleki-Ghaleh. H et al. reported that in this weak electromagnetic field of cells, Fe_3_O_4_ magnetic materials improved cell growth and activity by generating magnetic fields to enhance cell communication [24]. When it was combined with the matrix, a large number of tiny magnetic fields were generated on the pores or the surface of the scaffold. According to previous studies, magnetic fields may affect the nucleation of protein crystals in the culture medium and the distribution of proteins in the cell membrane, accelerating the specific recognition of integrin proteins on the cell surface and adsorbing to the extracellular matrix proteins on the surface of the scaffold, thereby promoting cell adhesion and spread [29,51]. Meanwhile, magnetic field stimulation can activate calcium ion (Ca^2+^) channels on the cell membrane, which can increase the concentration of calcium ions in cells, thereby improving the function of Ca^2+^/calmodulin and the activity of cyclin-dependent kinase, promoting the proliferation of osteoblasts [52,53,54].

In addition, the magnetic field can also activate various signal pathways of the cell, and collaboratively mediate the signal communication between them, such as the classic mitogen-activated protein kinase [55,56,57] and BMP signal pathway [17,58], thereby promoting the expression of growth factors, improving the activity of runt-related transcription factor 2 and ALP, accelerating the growth and differentiation of osteoblasts, and promoting bone repair finally. Of course, the micro magnetic force generated in the microenvironment of the magnetic scaffold can provide continuous dynamic mechanical stimulation to MG63 cells, which can also improve the cells adhere and migrate. In addition, the Fe_3_O_4_ nanoparticles in the scaffold have a large surface area to volume ratio. Uniform dispersion in the scaffold may show more contact surface area, thus providing more attachment sites for cell attachment [59]. However, the Fe_3_O_4_ nanoparticles are nanomaterials, and previous studies have indicated that the nanomaterials may cause some potential adverse effects on cells and organs of the human body [60,61,62]. For example, Long et al. [60] investigated the effect of Fe_3_O_4_ nanoparticles on cell activity of human hepatoma cell and lung adenocarcinoma cell, and their results indicated that the higher concentration of Fe_3_O_4_ nanoparticles would cause cell death. PLLA is a biodegradable and biocompatibility bone scaffold material which has a relatively low degradation rate [63,64]. When the Fe_3_O_4_ nanoparticles are incorporated into the PLLA scaffold, the slow degradation of scaffold can play the role of controlled release of Fe_3_O_4_ nanoparticles, thereby continuously and steadily enhancing cellular activity without causing adverse effects to human body.

## 4. Conclusions

In this study, PLLA/Fe_3_O_4_ scaffolds were successfully fabricated by SLS. The incorporated Fe_3_O_4_ nanoparticles not only enhance the mechanical properties of the PLLA scaffold, but also effectively improve the biological activity of the scaffold. The results showed that the PLLA/7%Fe_3_O_4_ composite scaffold exhibited increased compressive strength, modulus, and Vickers hardness, which were 29.1 MPa, 2.9 GPa, and 67.7 HV, respectively. Furthermore, the magnetic composite scaffold not only promoted cell attachment, diffusion, and interaction, but also significantly promoted MG63 cell proliferation and stimulated cell differentiation. All these positive results suggested that the SLS-processed PLLA/Fe_3_O_4_ scaffold was of great potential for bone regeneration.
